# A New Mouse Model for Complete Congenital Stationary Night Blindness Due to *Gpr179* Deficiency

**DOI:** 10.3390/ijms22094424

**Published:** 2021-04-23

**Authors:** Elise Orhan, Marion Neuillé, Miguel de Sousa Dias, Thomas Pugliese, Christelle Michiels, Christel Condroyer, Aline Antonio, José-Alain Sahel, Isabelle Audo, Christina Zeitz

**Affiliations:** 1 Institut de la Vision, Institut National de la Santé et de la Recherche Médicale, Centre National de la Recherche Scientifique, Sorbonne Université, F-75012 Paris, France; elise.orhan@yahoo.fr (E.O.); marion.neuille@gmail.com (M.N.); migueldias670@hotmail.com (M.d.S.D.); pugliese.thomas@gmail.com (T.P.); christelle.michiels@inserm.fr (C.M.); christel.condroyer@inserm.fr (C.C.); aline.antonio@inserm.fr (A.A.); j.sahel@gmail.com (J.-A.S.); isabelle.audo@inserm.fr (I.A.); 2 Centre Hospitalier National d’Ophtalmologie des Quinze-Vingts, INSERM-DGOS CIC1423, F-75012 Paris, France; 3 Fondation Ophtalmologique Adolphe de Rothschild, F-75019 Paris, France; 4 Academie des Sciences, Institut de France, F-75006 Paris, France; 5 Department of Ophthalmology, The University of Pittsburgh School of Medicine, Pittsburgh, PA 15213, USA; 6 Institute of Ophthalmology, University College of London, London EC1V 9EL, UK

**Keywords:** congenital stationary night blindness, cCSNB, GPR179, mouse model, b-wave, optomotor responses, dendritic tip staining, ON-bipolar cells, retina

## Abstract

Mutations in *GPR179* lead to autosomal recessive complete congenital stationary night blindness (cCSNB). This condition represents a signal transmission defect from the photoreceptors to the ON-bipolar cells. To confirm the phenotype, better understand the pathogenic mechanism in vivo, and provide a model for therapeutic approaches, a *Gpr179* knock-out mouse model was genetically and functionally characterized. We confirmed that the insertion of a neo/lac Z cassette in intron 1 of *Gpr179* disrupts the same gene. Spectral domain optical coherence tomography reveals no obvious retinal structure abnormalities. *Gpr179* knock-out mice exhibit a so-called no-b-wave (*nob*) phenotype with severely reduced b-wave amplitudes in the electroretinogram. Optomotor tests reveal decreased optomotor responses under scotopic conditions. Consistent with the genetic disruption of *Gpr179*, GPR179 is absent at the dendritic tips of ON-bipolar cells. While proteins of the same signal transmission cascade (GRM6, LRIT3, and TRPM1) are correctly localized, other proteins (RGS7, RGS11, and GNB5) known to regulate GRM6 are absent at the dendritic tips of ON-bipolar cells. These results add a new model of cCSNB, which is important to better understand the role of GPR179, its implication in patients with cCSNB, and its use for the development of therapies.

## 1. Introduction

The first steps in vision occur in the mammalian retina when rod and cone photoreceptors transform light into a biochemical signal, which is processed by bipolar cells. Synaptic transmission between photoreceptors and ON-bipolar cells is mediated by the neurotransmitter glutamate, which is released by photoreceptors and binds to the metabotropic glutamate receptor GRM6 (also called mGluR6) [1]. In darkness, glutamate is spontaneously released in the synaptic cleft which stimulates GRM6 and in turn activates its trimeric G protein and produces activated Gαo-GTP and free Gβγ [2]. Subsequently, activated Gαo-GTP inhibits TRPM1 cation channel opening. Upon light stimulation, glutamate release is reduced at the synaptic cleft, which is sensed via GRM6 by ON-bipolar cells. This leads to a reduction in Gαo activation and to TRPM1 opening, resulting in ON-bipolar cell depolarization [3,4,5]. NYX and LRIT3 are suggested to both be important for the correct localization of TRPM1 at the dendritic tips of ON-bipolar cells [6,7,8,9]. After their activation by their GPCR, such as GRM6, G proteins spontaneously deactivate at a slow rate. They require assistance of regulator of G protein signaling (RGS) proteins to inactivate them by increasing the rate of GTP hydrolysis of the G protein [10]. RGS7, RGS11, and guanine nucleotide-binding protein subunit beta-5 (GNB5) have been implicated in the complex that regulates Gαo deactivation in ON-bipolar cell cascade, and all co-localize at the dendritic tips of ON-bipolar cells [11,12,13]. The specificity of RGS proteins depends on the formation of macromolecular complexes with other proteins that dictate their compartmentalization. Two homologous membrane anchoring subunits have been shown to form complexes with R7 RGS proteins and increase the activity of RGS proteins by targeting them at the plasma membrane: the regulator of G protein signaling 9 binding protein (RGS9BP) and the regulator of G protein signaling 7 binding protein (RGS7BP) [14,15,16,17].

Patients with a functional signaling defect from photoreceptors to ON-bipolar cells present a congenital stationary night blindness (CSNB) phenotype. CSNB comprises a group of clinically and genetically heterogeneous retinal disorders. Patients often complain of night or dim light vision disturbance or delayed dark adaptation. Poor visual acuity, myopia, nystagmus, strabismus, and fundus abnormalities are other ophthalmic signs that can be reported. Vision under dark adaptation is rarely tested routinely and CSNB is likely overlooked by clinicians, underestimating its prevalence [18]. Data from our French cohort estimates that it is a rare disease with a prevalence of at least 1:400,000 affected patients [19]. The underestimation of its prevalence may be due to the requirement for specific clinical tests to correctly diagnose CSNB. While the fundus of patients with CSNB is normal or may show only myopic changes with marginal changes on spectral domain optical coherence tomography (SD-OCT), full-field electroretinogram (ERG) recordings show specific alterations. Thus, ERG recordings are critical for functional phenotyping and precise diagnosis [20]. Most of the patients present dysfunction of signal transmission between photoreceptors and bipolar cells and show a characteristic Schubert–Bornschein ERG response, with normal scotopic a-wave and severely reduced b-wave leading to an electronegative waveform [21]. The Schubert–Bornschein type of ERG can be classified into two subgroups: a complete (cCSNB) and incomplete form (icCSNB) showing ON- or both ON- and OFF-bipolar pathway dysfunction, respectively [22]. cCSNB is characterized by a drastically reduced rod-driven b-wave due to selective ON-bipolar cell dysfunction and specific cone ERG waveforms [23]. cCSNB has been associated with mutations in *NYX* (MIM# 300278) [24,25], *GRM6* (MIM# 604096) [26,27], *TRPM1* (MIM# 603576) [28,29,30], *GPR179* [MIM# 614515]([31,32]), and *LRIT3* (MIM# 615004) [8]. These genes are expressed in the inner nuclear layer (INL) and encode proteins localized at the dendritic tips of ON-bipolar cells [5,6,7,8,30,32,33,34,35,36,37,38,39] implicated in signaling from photoreceptors to bipolar cells. Recent findings in mice suggested that *Lrit3* is expressed presynaptically [40]. The mutation spectrum of those genes underlying cCSNB includes all possible variants [20]. More specifically, today more than twenty different disease-causing *GPR179* variants have been described encompassing missense, nonsense, splice-site variants, small insertions and deletions or insertion–deletions, a gross deletion, and a deep intronic variant predicted to influence splicing (The Human Gene Mutation Database (HGMD^®^), April 2021) [20,41,42]. Truncating variants are all predicted to lead to loss of function of the respective protein, and also studies including immunolocalization studies for missense variants and splice-site assays suggest that loss of function is the common underlying pathogenic mechanism in *GPR179*-related cCSNB and in cCSNB in general [34,42]. This is furthermore underpinned by the uniform phenotype observed in patients with cCSNB [20].

Animal models are an excellent tool for identifying and elucidating the pathogenic mechanisms of gene defects underlying cCSNB. The phenotypes of these models can be assessed in patients by performing full-field ERG, SD-OCT in vivo, and by post mortem studies [20,43]. Various animal models have been designed or naturally occur for cCSNB with dysfunction in molecules important for the signaling from the photoreceptors to the adjacent bipolar cells [3,4,5,6,9,32,33,35,40,43,44,45,46,47,48,49,50,51,52,53,54]. Mutation independent, the pathogenic mechanism is characterized by loss of protein function leading to a stationary phenotype, characterized by a dysfunction under scotopic conditions with absent b-waves on the ERG responses and no obvious morphological abnormalities. Nevertheless, cCSNB mouse models have more severely affected photopic b-waves than in humans (Varin et al., 2021, under revision). in particular, mouse models were helpful in dissecting the ON-bipolar cell signaling cascade [20]. When our group identified mutations in patients with cCSNB in *GPR179* coding for a functionally unknown orphan receptor, there was no corresponding mouse model [31]. To validate our findings in a mouse model and dissect the role of GPR179, we initiated this project. Independently, another group identified mutations in *GPR179* in patients with cCSNB and provided another mouse model with this gene defect, *Gpr179^nob5^* [32]. Therapeutic approaches using mice lacking functional NYX, GRM6, and LRIT3 partially restored the cCSNB phenotype [40,55,56] (Varin et al., 2021, under revision). Here, we describe the generation and characterization of a novel *Gpr179*^−/−^ mouse model which allows the study of the gene defect in an animal model to investigate the role of GPR179 in the signaling cascade in respect to other molecules implicated herein and which delivers the basis to study therapeutic approaches.

## 2. Results

### 2.1. Design and Genotyping of the Gpr179 KO First Mouse Model

**Figure 1:**
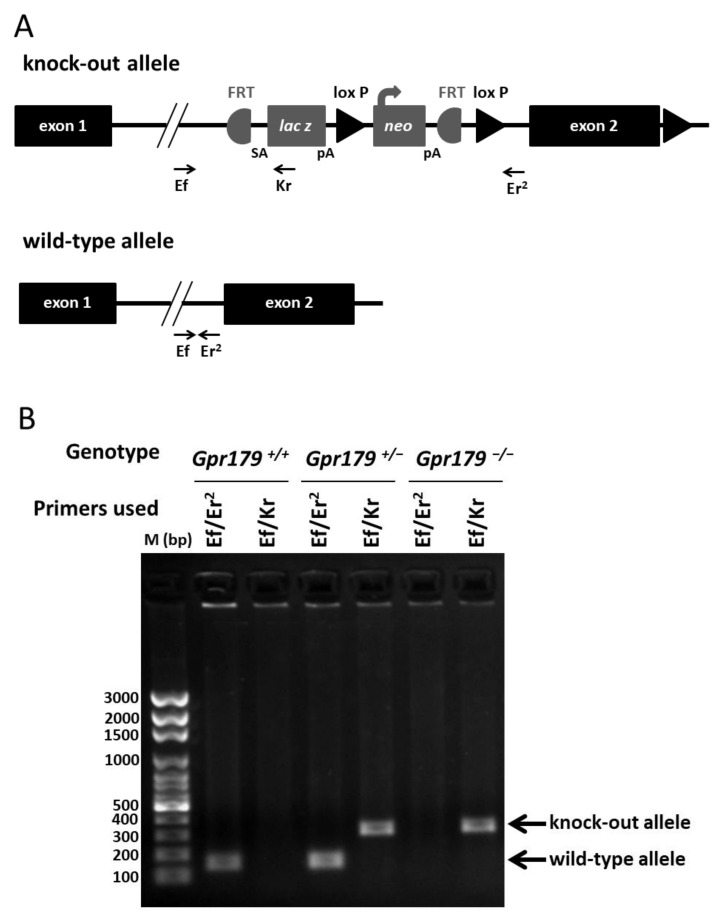
(**A**) Schematic drawing of the wild-type allele and the knock-out allele with the construction inserted in intron 1. Abbreviations: FRT: flippase recognition target, SA: signal anchor, *lacZ*: lactose operon, pA: polyadenylation site, *neo*: neomycin. M bp: marker base pair. For genotyping, *mGpr179*_Ef (Ef) and *mGpr179*_Er2 (Er2) primers were designed to amplify a 146 bp product on wild-type allele. Ef and *mGpr179*_Kr (Kr) primers were designed to amplify a 303 bp product on knock-out allele. (**B**) After migration on 1% agarose gel, *Gpr179*^+/+^ exhibited a single fragment at the expected size of 146 bp, *Gpr179*^−/−^ exhibited a single fragment at the expected size of 303 bp, and *Gpr179*^+/−^ exhibited both fragments.

To further investigate the role of GPR179 in vivo, we created a *Gpr179* knock-out mouse. For the mutant allele, a cassette containing *lacZ* and *neo* was inserted in intron 1 of *Gpr179* (Figure 1A). Since *lacZ* contains a termination codon, this construction is predicted to lead to a premature termination codon and also disruption of the protein. Prior to the study, we genotyped the founder animals for mutations in known mouse genes underlying cCSNB and common mutations present in commercially available mouse lines. We did not find any mutation, except the homozygous *Crb1/rd8* mutation, which was already described in the C57Bl6/N strain [57] used for ES-clones. We backcrossed two generations of these animals with C57Bl6/J mice that were free from the *Crb1/rd8* mutation and obtained heterozygous knock-out mice for *Gpr179* free from any other mutation in the screened genes. The heterozygous knock-out mice for *Gpr179* were intercrossed to produce wild-type (*Gpr179*^+/+^), heterozygous (*Gpr179*^−/+^), and mutant (*Gpr179*^−/−^) offspring. Genotypic conditions (see material and methods) were established to distinguish wild-type (*Gpr179*^+/+)^, heterozygous (*Gpr179*^−/+^), and mutant (*Gpr179*^−/−^) offspring (Figure 1B).

### 2.2. Structural Characterization by SD-OCT

**Figure 2:**
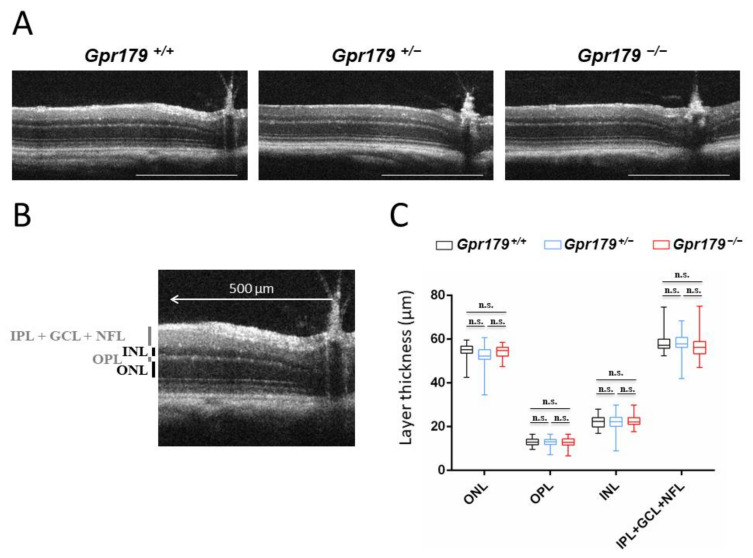
Spectral domain optical coherence tomography (SD-OCT) retinal morphology and layer thicknesses of 3-month-old *Gpr179*^+/+^, *Gpr179*^+/−^, and *Gpr179*^−/−^ mice. (**A**) Representative SD-OCT sections of 3-month-old *Gpr179*^+/+^, *Gpr179*^+/−^, and *Gpr179*^−/−^ mice. Scale bar: 500 µm. (**B**) Outer nuclear layer (ONL), outer plexiform layer (OPL), inner nuclear layer (INL), a complex comprising inner plexiform layer (IPL), ganglion cell layer (GCL), and nerve fiber layer (NFL) called IPL+GCL+NFL are measured at 500 μm of the optic nerve on SD-OCT images. (**C**) ONL, OPL, INL, and IPL + GCL + NFL thicknesses of 3-month-old *Gpr179*^+/+^ (black box), *Gpr179*^+/−^ (blue box), and *Gpr179*^−/−^ (red box) mice were compared. Error bars represent standard errors; n.s. indicates a non-significant test (*p* > 0.05).

SD-OCT was performed on 10 *Gpr179*^+/+^, 12 *Gpr179*^−/+^, and 10 *Gpr179*^−/−^ at 3 months of age. We compared the retinal morphology for *Gpr179*^+/+^, *Gpr179*^−/+^, and *Gpr179*^−/−^ mice, and we did not notice any obvious modification (Figure 2A). ONL, OPL, INL, IPL + GCL + NFL thicknesses were measured (Figure 2B), and no changes were observed in layer thickness in any quadrant (dorsal, ventral, temporal, or nasal) for each animal; therefore, these values were combined. No statistical difference was observed in the different layer thicknesses across the three genotypes (Figure 2C).

### 2.3. Functional Characterization

**Figure 3:**
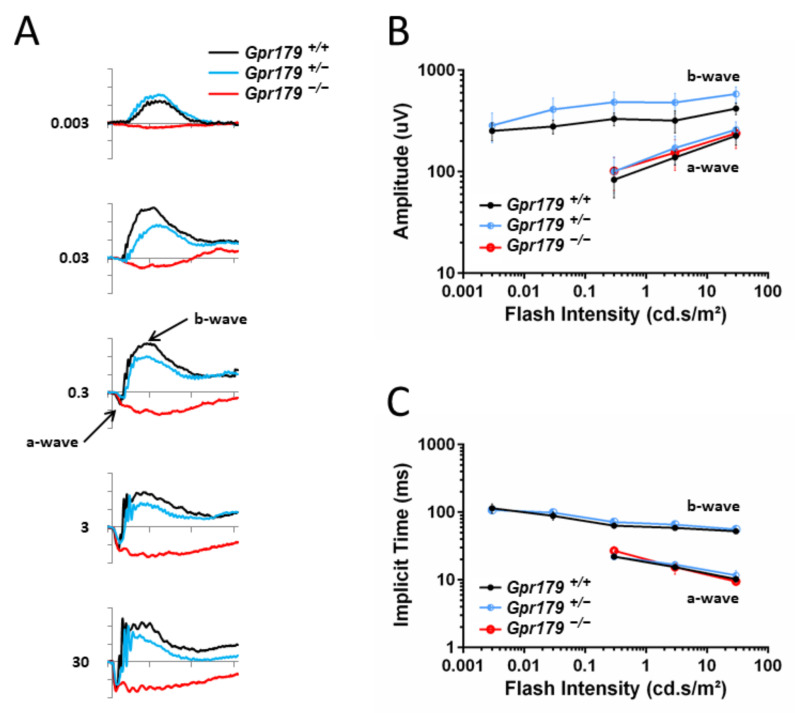
Scotopic electroretinogram, (ERG) responses. Dark-adapted ERG series were obtained from 10 *Gpr179*^+/+^ (black line), 12 *Gpr179*^+/−^ (blue line), and 10 *Gpr179*^−/−^ (red line) littermates. (**A**) Representative waveforms as a function of stimulation intensity. The scale marks indicate 100 ms (time in abscissa) and 200 µV (amplitude in ordinate). Values to the left of the waveforms indicate stimulation flash intensity in cd.s/m^2^. a-waves and b-waves are depicted with arrows for the *Gpr179*^+/+^ mouse recording at 0.3 cd.s/m^2^. Amplitude (**B**) and implicit time (**C**) of the major components of the dark-adapted ERG with increasing flash intensity. The b-wave component is absent in *Gpr179*^−/−^ mice, and therefore these data are not plotted.

Recording of the ERG was performed on 10 *Gpr179*^+/+^, 12 *Gpr179*^−/+^, and 10 *Gpr179*^−/−^ at 3 months of age. Under scotopic conditions, which allow the testing of the rod pathway function, *Gpr179*^+/+^ showed normal responses for both a- and b-wave. As expected, amplitudes of both a- and b-waves increased with increasing flash intensities (Figure 3A), and the implicit times of both a- and b-waves decreased (Figure 3B). The ERG responses of *Gpr179*^+/−^ heterozygous mice were similar to the *Gpr179*^+/+^ ERG responses (Figure 3A,B). In contrast, *Gpr179*^−/−^ mice were lacking b-waves on their ERG responses, while a-waves were comparable in amplitude or implicit time to *Gpr179*^+/+^ and *Gpr179*^+/−^, leading to an electronegative ERG waveform (Figure 3A–C). These results indicate a signal transmission defect between rod photoreceptors and ON-bipolar cells, whereas the phototransduction in rod photoreceptors is not affected.

**Figure 4:**
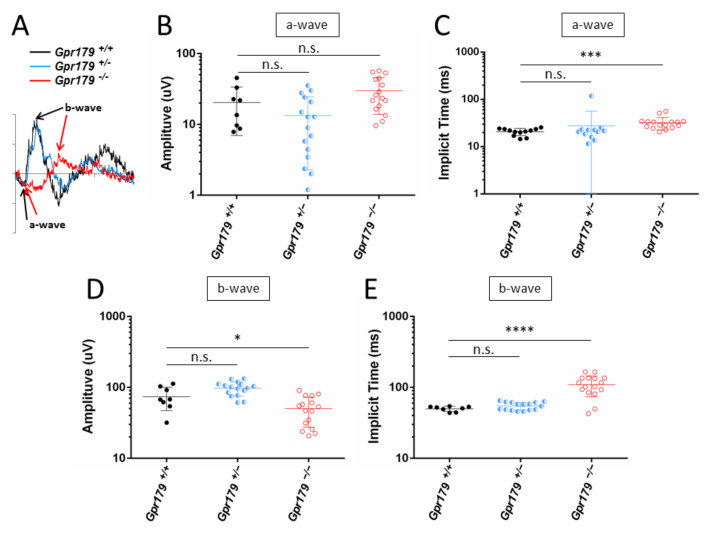
Photopic electroretinogram (ERG) responses. Light-adapted ERG recordings with a stimulus intensity of 3 cd.s/m^2^ were obtained from 10 *Gpr179*^+/+^ (black line), 12 *Gpr179*^+/−^ (blue line), and 10 *Gpr179*^−/−^ (red line) littermates. (**A**) Representative waveforms. The scale marks indicate 100 ms (time in abscissa) and 50 µV (amplitude in ordinate). a-waves and b-waves are depicted with black arrows for the *Gpr179*^+/+^ mouse recording, and red arrows for the *Gpr179*^−/−^ mouse recording. Amplitude (**B**) and implicit time (**C**) of the a-wave. Amplitude (**D**) and implicit time (**E**) of the b-wave. Abbreviations: n.s. = non-significant, *: *p* < 0.05, ***: *p* < 0.001, ****: *p* < 0.0001.

Under photopic conditions, which allow testing the cone-pathway function, the recordings were more variable. The *Gpr179*^+/+^ mice showed normal ERG responses for both a- and b-waves. No difference was observed in the a- and b-wave amplitudes and the implicit times of *Gpr179*^+/−^ mice when compared to *Gpr179*^+/+^ mice (Figure 4A–E). In contrast, the *Gpr179*^−/−^ mice showed delayed a-wave implicit time (Figure 4C), a decreased b-wave amplitude (Figure 4D), and delayed b-wave implicit times (Figure 4E). These results are in keeping with cone-mediated pathway dysfunction in these mice.

**Figure 5:**
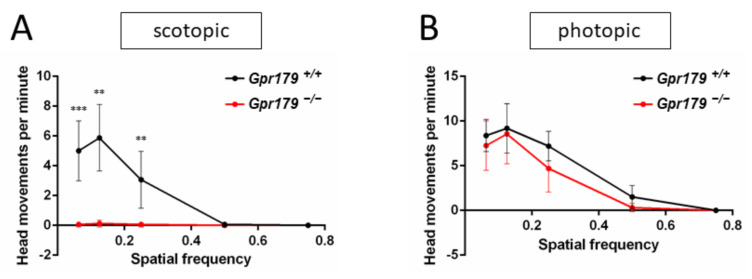
Optomotor responses. The number of head movements per minute was obtained in scotopic conditions (**A**) with spatial frequencies from 0.063 to 0.75 cycles per degree (cpd) and compared using the Kruskal–Wallis statistical test in representative *Gpr179*^+/+^ (black line) and *Gpr179*^−/−^ (red line) littermates (**B**). Abbreviations: **: *p* < 0.005, ***: *p* < 0.0012.4. Localization of the Proteins of the Cascade.

Optomotor tests were performed on 8 *Gpr179*^+/+^ and 8 *Gpr179*^−/−^ at 3 months of age under scotopic and photopic conditions at increasing spatial frequencies. Under scotopic conditions, a maximum number of head movements per minute was reached at 0.125 cpd for *Gpr179*^+/+^ mice. The number of head movements per minute decreased with increasing spatial frequency, reaching zero at 0.5 cycles per degree (cpd) (Figure 5A). For the *Gpr179*^−/−^ mice, the optomotor responses were strongly decreased to almost zero for all spatial frequencies (Figure 5A), indicating a dysfunction under scotopic conditions. Under photopic conditions, a maximum number of head movements per minute was reached at 0.125 cpd for the *Gpr179*^+/+^ mice. The number of head movements per minute decreased with increasing spatial frequency, reaching zero at 0.75 cpd (Figure 5B). When compared to the *Gpr179*^+/+^ mice, no statistical difference was observed for the *Gpr179*^−/−^ mice (Figure 5B), indicating no obvious dysfunction in photopic conditions for these mice.

**Figure 6:**
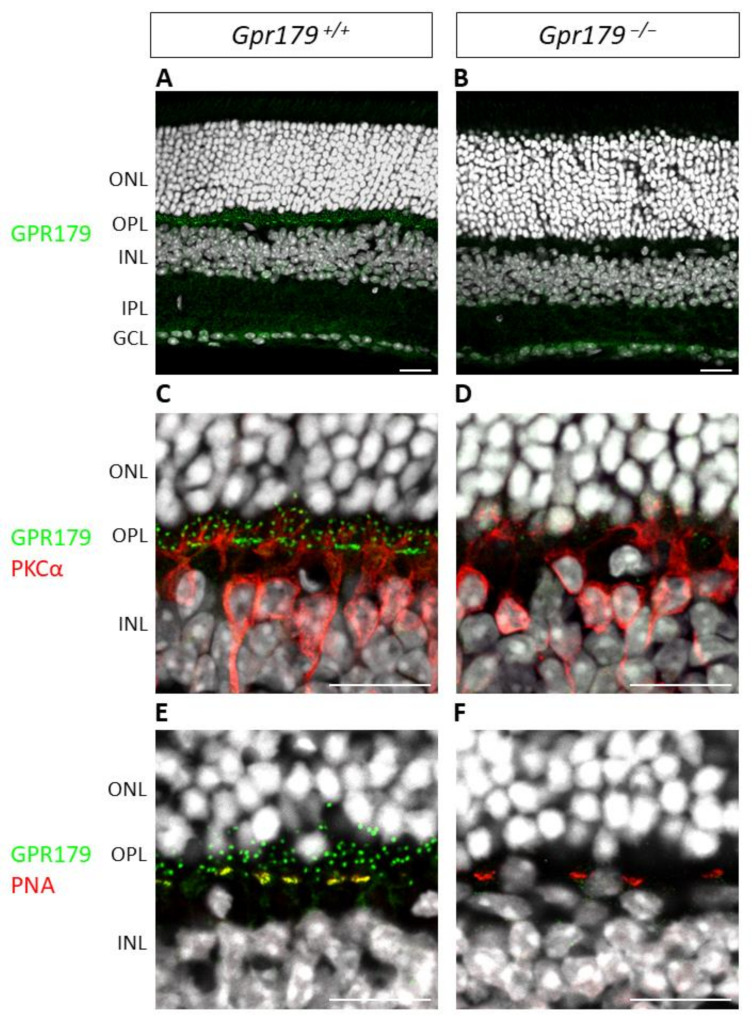
Validation of *Gpr179* knock-out model. Representative confocal images of *Gpr179*^+/+^ and *Gpr179*^−/−^ mouse retinal sections. *Gpr179*^+/+^ (**A**) and *Gpr179*^−/−^ (**B**) mouse retinal sections were stained with anti-GPR179 (green) and DAPI (white). (**C**,**D**) are shown with a 4× zoom focused on the outer plexiform layer (OPL) of (**A**,**B**), with additional staining of rod ON-bipolar cell specific marker PKCα (red). (**E**,**F**) are focused images of OPL of *Gpr179*^+/+^ (**E**) and *Gpr179*^−/−^ (**F**) mouse retinal sections stained with anti-GPR179 (green), anti-PNA which is a marker of cone pedicle (red), and DAPI (white). Scale bar: 10 µm, ONL: outer nuclear layer; OPL: outer plexiform layer; INL: inner nuclear layer; IPL: inner plexiform layer; GCL: ganglion cell layer.

To validate our *Gpr179* knock-out model, we performed immunohistochemistry to confirm that the GPR179 protein was absent in *Gpr179*^−/−^ mice. Immunohistochemistry of the *Gpr179*^+/+^ mouse retinal sections revealed a clear staining in the OPL (Figure 6A,C,E), which was absent in *Gpr179*^−/−^ (Figure 6B,D,F). Some slight non-specific staining remained in the IPL and the GCL in both the *Gpr179*^+/+^ and *Gpr179*^−/−^ mice (Figure 6A–B). In the *Gpr179*^+/+^ mouse retinal sections, the staining was specifically found at the dendritic tips of presumably all ON-bipolar cells (Figure 6C,E), co-stained for the specific marker of rod ON-bipolar cells PKCα (Figure 6C) and for the cone pedicle specific marker PNA (Figure 6E). This specific GPR179 staining was absent in the *Gpr179*^−/−^ mouse retinal sections, while PKCα and PNA staining was unaffected (Figure 6B,D,F).

**Figure 7:**
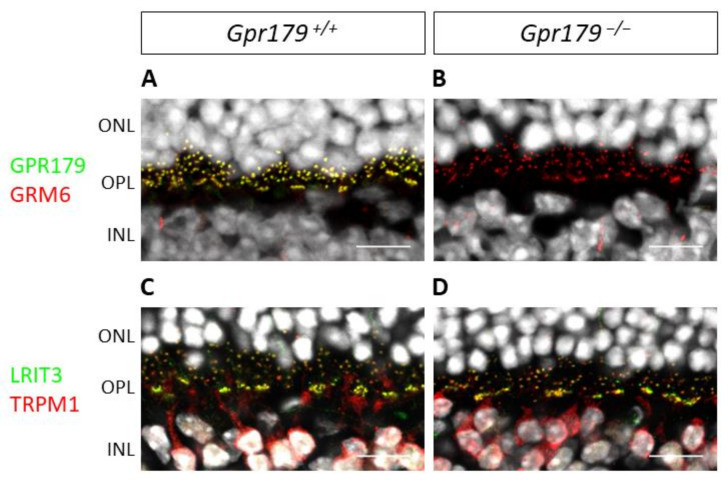
Localization of GRM6, TRPM1, and LRIT3 at the dendritic tips of ON-bipolar cells is independent of Gpr179 expression. Representative confocal images of *Gpr179*^+/+^ and *Gpr179*^−/−^ mouse retinal sections. *Gpr179*^+/+^ (**A**) and *Gpr179*^−/−^ (**B**) mouse retinal sections were stained with anti-GPR179 (green), anti-GRM6 (red), and DAPI (white). *Gpr179*^+/+^ (**C**) and *Gpr179*^−/−^ (**D**) mouse retinal sections were stained with anti-LRIT3 (green), anti-TRPM1 (red), and DAPI (white). Scale bar: 10 µm, ONL: outer nuclear layer; OPL: outer plexiform layer; INL: inner nuclear layer.

Once the model had been validated by immunohistochemistry, we studied the localization of various proteins involved in this ON-bipolar signaling cascade. The immunohistochemistry of the *Gpr179*^−^/^−^ mouse retinal sections revealed ON-bipolar cell dendritic tip staining for GRM6 (Figure 7B), TRPM1, and LRIT3 (Figure 7D), similar to findings in the *Gpr179*^+/+^ mouse retinal sections (Figure 7A,C).

**Figure 8:**
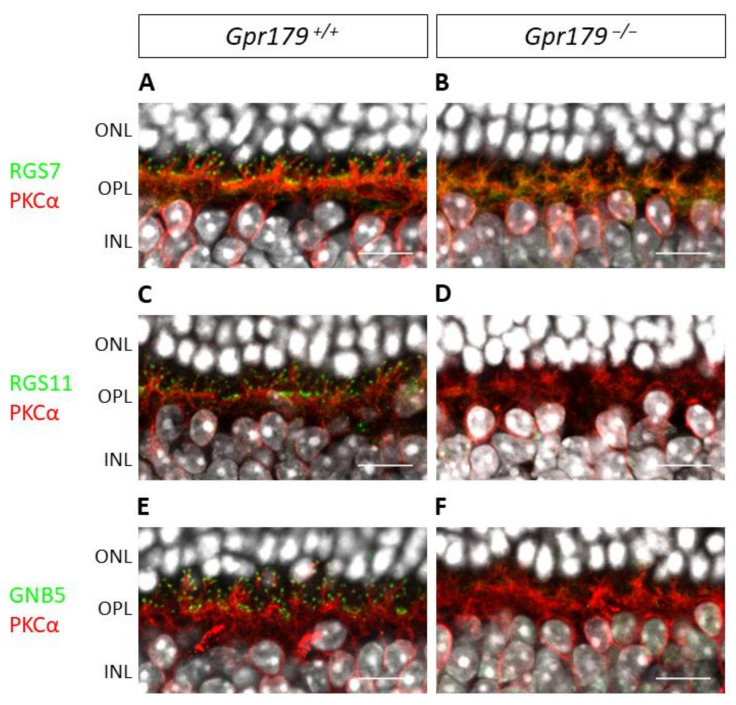
Localization of RGS11, RGS7, and GNB5 at the dendritic tips of ON-bipolar cells is dependent on Gpr179 expression. Representative confocal images of *Gpr179*^+/+^ and *Gpr179*^−/−^ mouse retinal sections. *Gpr179*^+/+^ (**A**) and *Gpr179*^−/−^ (**B**) mouse retinal sections were stained with anti-RGS7 (green), anti-PKCα (red), and DAPI (white). *Gpr179*^+/+^ (**C**) and *Gpr179*^−/−^ (**D**) mouse retinal sections were stained with anti-RGS11 (green), anti-PKCα (red), and DAPI (white). *Gpr179*^+/+^ (**E**) and *Gpr179*^−/−^ (**F**) mouse retinal sections were stained with anti-GNB5 (green), anti-PKCα (red), and DAPI (white). Scale bar: 10 µm, ONL: outer nuclear layer; OPL: outer plexiform layer; INL: inner nuclear layer.

However, the RGS proteins of GRM6 showed a different profile (Figure 8). The immunohistochemistry of the *Gpr179*^+/+^ mouse retinal sections against RGS7, RGS11, GNB5, and co-stained for the specific marker of ON-bipolar cells PKCα revealed the localization of these proteins at the dendritic tips of bipolar cells (Figure 8A,C,E), whereas there was no staining on *Gpr179*^−/−^ mouse retinal sections (Figure 8B,D,F).

## 3. Discussion

In this work, we delivered a new *Gpr179* knock-out mouse model which we characterized genetically, structurally by SD-OCT and functionally by ERG recordings and optomotor measurements. To better understand the role of GPR179 in the retina and localize it in this signaling cascade, the localization of other proteins of the signaling cascade was investigated by immunohistochemistry. This *Gpr179* knock-out mouse model carried a cassette inserted in intron 1 of *Gpr179*, predicted to lead to a mutated protein with amino acids encoded by exon 1 of *Gpr179* but also the entire *lacZ* disrupting *Gpr179* (Figure 1). Retinal thickness monitoring of the *Gpr179* knock-out mouse was investigated using high resolution imaging with SD-OCT (Figure 2). Indeed, this non-invasive in vivo technique enables the investigation of the retinal morphology without the terminal nature of histology [58,59]. Herein, we showed that retinal thickness revealed no modification in this mouse model. As far as we know, no severe retinal morphological modification was described for any other mouse models of cCSNB [4,35,46,47,48,49,51,54] when investigated by histology, whereas SD-OCT revealed thinning of the inner nuclear layer and part of the retina containing the inner plexiform layer, ganglion cell layer, and nerve fiber layer in the *Lrit3* knock-out mouse model (*nob6*) [43]. This difference is likely to be due to a function of LRIT3 in synapse formation [60]. Furthermore, functional characterization of this *Gpr179* knock-out mouse model revealed a *nob* phenotype as observed under scotopic conditions in patients with cCSNB due to *GPR179* mutations [31,32]. Indeed, *Gpr179*^-/-^ mice present a Schubert–Bornschein-type ERG with a normal a-wave and severely reduced b-wave under scotopic conditions (Figure 3). The preserved a-wave indicates that rod photoreceptors respond normally to light, and the lack of the b-wave localizes the defect to synaptic transmission from rod photoreceptors to the rod ON-bipolar cells. In photopic conditions, the responses were in general more variable. Nevertheless, cone-mediated pathways were also severely affected: the implicit times of both, a- and b-waves were delayed, and the amplitude of the b-wave was severely reduced. Therefore, visual dysfunction in *Gpr179*^−/−^ mice affects both rod- and cone-ON-bipolar systems. Optomotor tests revealed that the number of head movements was dramatically decreased under scotopic conditions, while no statistical difference was observed in photopic conditions. Our results were consistent with another *Gpr179* mutant mouse line harboring a *Gpr179* transposon mutation (*nob5*) and a morpholino knockdown of *Gpr179* in zebrafish [32], as well as with other mice with mutations in genes implicated in the retinal ON pathway, most strikingly lacking the scotopic ERG b-wave [20]. Note that regular scotopic ERG measurements are recorded for 300 ms. When recorded for longer, Ray et al. showed on *nob5* mice a small b-wave-like response with a longer implicit time in response to regular low luminance flashes (−3.6 to −2.4 log cd.s/m^2^), this b-wave amplitude being increased with longer flash duration [61]. Here, the recording conditions did not allow confirming the existence of this b-wave on *Gpr179*^−/−^ mice. However, it needs to be noted that in general the cCSNB phenotype in mice seems to affect more severely the cone-mediated pathway compared to patients with cCSNB as measured by ERG recordings (Varin et al., 2021 under revision). Immunohistochemistry directed against GPR179 on the new *Gpr179*^−/−^ mouse model confirmed that our mouse was indeed a knock-out for GPR179 since we could not observe the specific dendritic tip staining (Figure 6). The immunolocalization of GPR179 partners was also consistent with results obtained on the other *Gpr179* mouse model [32,39,61,62,63]. On one hand, GRM6 [32,61], TRPM1 [39,61], and LRIT3 are not mislocalized when GPR179 is lacking (Figure 7). On the other hand, GPR179 disruption leads to the absence at the dendritic tips of ON-bipolar cells of RGS7 [39], RGS11 [39], and GNB5 (Figure 8). Proteomic screening identified GPR179 as a binding partner of RGS proteins [39]. Together these findings suggest that GPR179 has a role in targeting and/or maintaining either RGS7-GNB5 or RGS11-GNB5 complexes at the dendritic tips of ON-bipolar cells. It is also known that GPR179 forms macromolecular complexes with GRM6 and TRPM1 [62], suggesting a role in the compartmentalization of the principal elements of the cascade by bringing RGS proteins into close proximity to key elements of the cascade they regulate. Our studies showed that the localization of various proteins involved in ON-bipolar signal transmission including LRIT3 is maintained, whereas proteins of the RGS family, which are known to regulate GRM6 [15], are no longer localized at the dendritic tips of ON-bipolar cells. Similar to our observations in *Gpr179*^−/−^ mice, GRM6 and TRPM1 were correctly localized at the dendritic tips of ON-bipolar cells in a double knock-out model for RGS7 and RGS11 (DKO) [64] and in the *nob5* mouse [61]. In DKO, single-cell recordings of a light-evoked current for scotopic stimulation were performed on rod ON-bipolar cells and revealed no detectable current when stimulated briefly. In contrast, when the stimulation was brighter and longer (20 s), a small and delayed current, compared to the wild-type, was recorded. This suggests that GRM6 and its cascade maintain the capacity to close TRPM1 channel after a light stimulation, and that RGS7 and RGS11 are implicated in the sensitivity and the time course of the ON-bipolar cell responses by acting on the inactivation of Gαo [64]. In the *nob5* model, a pharmacological approach was used when performing a single-cell patch clamp [61] on rod ON-bipolar cells. The responses of DKO and *nob5* to puffs of cyclopropyl-4-phosphonophenylglycine (CPPG), which is a GRM6 antagonist and indirectly closes TRPM1, are similar to the wild-type response but smaller in amplitude, with *nob5*′s amplitude being superior to DKO’s amplitude. TRPM1 can also be closed even without GPR179 and RGS proteins. The responses of DKO and *nob5* to capsaicin, which is a TRPM1 agonist and closes TRPM1, are similar to the wild-type response but smaller in amplitude, with *nob5′s* amplitude being inferior to DKO’s amplitude. GPR179 also allows the TRPM1 channel to be closed by capsaicin and is required in the sensitivity to TRPM1′s closure [61]. Together, these results suggest a role of GPR179 in the sensitivity of the GRM6 signaling cascade with a role in the TRPM1 channel. Together, we delivered herein a novel mouse model mimicking the phenotype of patients with cCSNB due to mutations in *GPR179,* which delivers the basis to better understand the pathophysiology of the disease and develop therapeutic approaches to treat cCSNB. Indeed, partial restoration of the proteins underlying cCSNB and the phenotype was obtained using AAV-mediated gene therapy approaches for mice lacking *Nyx*, *Lrit3,* and *Grm6* [40,55,56] (Varin, 2021, under revision). However, the bottleneck of an AAV-gene therapy approach for mice lacking *Gpr179* will be the larger size of the coding sequence of *Gpr179* (>6 kb), making it too large to pack it into an AAV. Different research groups have tested the use of dual and triple AAV vector systems; however, this technique may need to be well established to obtain sufficient protein expression for successful treatment [65].

## 4. Materials and Methods

### 4.1. Animal Care

All animal procedures were performed according to the Association for Research in Vision and Ophthalmology (ARVO) Statement for the Use of Animals in Ophthalmic and Visual Research. *Gpr179^tm1a(KOMP)Mbp^* embryonic stem cells (ESCs) from agouti C57BL6/N mice were obtained from the Knock-Out Mouse Project (KOMP, Davis, CA, USA) Repository (https://www.mmrrc.org/catalog/StrainCatalogSearchForm.php?SourceCollection=KOMP (accessed on 29 March 2021)). *Gpr179^tm1a(KOMP)Mbp^* ESCs were injected into blastocysts from non-agouti C57BL6/N females at the Mouse Clinical Institute (Illkirch, France), and chimeras were crossed with non-agouti C57BL6/N mice to obtain non-agouti C57BL6/N mice heterozygous for the *Gpr179* mutation. These mice were then crossed twice with C57BL6/J to eliminate the homozygous *rd8* mutation, present in the C57BL6/N strain. We subsequently rederived the mice (Charles River, Chatillon-sur-Chalaronne, France) in order to obtain a Specific Pathogen Free (SPF) sanitary status. Heterozygous knock-out mice for *Gpr179* were intercrossed (Centre d’Exploration et de Recherche Fonctionnelle Expérimentale CERFE, Evry, France) to produce wild-type (*Gpr179*^+/+^), heterozygous (*Gpr179*^−/+^), and mutant (*Gpr179*^−/−^) offspring. SD-OCT and ERG were performed on the same 10 *Gpr179*^+/+^, 12 *Gpr179*^−/+^, and 10 *Gpr179*^−/−^ at 3 months of age. Since the structure and function of *Gpr179*^+/+^ and *Gpr179*^+/−^ mice retinas were not distinguishable, we continued the experimentations only on *Gpr179*^+/+^ and *Gpr179*^−/−^ mice. Optomotor tests were performed on 8 *Gpr179*^+/+^ and 8 *Gpr179*^−/−^ at 3 months of age, different from the SD-OCT and ERG experiments. Immunohistochemistry experiments were performed on 2 *Gpr179*^+/+^ and 2 *Gpr179*^−/−^ at 4 months of age. Mice were housed in a temperature-controlled room with a 12-h light/dark cycle. Fresh water and rodent feed were available *ad libitum*.

### 4.2. Genotyping

#### 4.2.1. Polymerase Chain Reaction (PCR) Genotyping for Gpr179

DNA was extracted from mouse tails with 50 mM NaOH after incubation at 95 °C for 30 min. Wild-type and mutant alleles were amplified independently using a polymerase (HOT FIREPol, Solis Biodyne, Tartu, Estonia), the same forward primer (*mGpr179*_Ef, 5′ CTGCCCCCACAGAATGTTCCCA3′), and two specific reverse primers: *mGpr179*_Er2 (5′CACCGCCTCTTTACTCTGCCCA3′) for the wild-type allele and *mGpr179*_Kr (5′ GGGCAAGAACATAAAGTGACCCTCC3′) for the mutant one, and the following program: 10 min at 95 °C for denaturation, 30 cycles of 45 s at 95 °C, 1 min at 60 °C, and 1 min at 72 °C, and for final extension 10 min at 72 °C. This gives rise to the following amplicons: PCR using mGpr179_Ef and *mGpr179*_Er2 primers amplifies a product of 146 base pairs (bp) for the wild-type allele and no product for the mutant allele; PCR using *mGpr179*_Ef and *mGpr179*_Kr primers amplifies no product for the wild-type allele and a 303 bp product for the mutant allele. PCR products were separated by electrophoresis on 1% agarose gels, stained with ethidium bromide, and visualized using a documentation system (Gel Doc XR+ system, Bio-Rad, Hercules, CA, USA) (Figure 1, Construction and validation of a novel Gpr179^−^/^−^).

#### 4.2.2. Genotyping for Common Mutations Found in Laboratory Mouse Strains

DNA of founder mice were used to genotype for the *Crb1/rd8*, *Pde6β/rd1*, and *Gnat2/cpfl3* mutations as previously described [43].

#### 4.2.3. Genotyping for Genes with Mutations Underlying cCSNB

DNA of founder mice were used to sequence the flanking intronic and exonic sequences of *Grm6*, *Gpr179*, *Nyx*, *Lrit3,* and *Trpm1,* as well as intron 2 of *Grm6* and intron 1 of *Gpr179* as previously described [43].

### 4.3. Spectral Domain Optical Coherence Tomography

Mice were anesthetized with ketamine (80 mg/kg) and xylazine (8 mg/kg) for ERG, and Spectral Domain Optical Coherence Tomography (SD-OCT) was performed on anesthetized animals, as previously described [43,59]. Briefly, the pupils were dilated with eye drops (0.5% mydriaticum, 5% neosynephrine), and SD-OCT images were recorded for both eyes using a spectral domain ophthalmic imaging system (Bioptigen, Inc., Durham, NC, USA). We performed rectangular scans consisting of a 1.4 mm by 1.4 mm perimeter with 1000 A-scans per B-scan with a total B-scan amount of 100. Scans were obtained first while centered on the optic nerve, and then with the nerve displaced either temporally/nasally or superiorly/inferiorly. SD-OCT scans were exported from InVivoVue as AVI files. These files were loaded into ImageJ (version 1.47; National Institutes of Health, Bethesda, MD, USA) where they were registered using the Stackreg plug-in. If the optic nerve was placed temporally/nasally, three B scans at the level of the nerve were averaged and measurements were performed 500 mm away from the optic disc on each side. In the case where the optic nerve was placed superiorly/inferiorly, 3 B-scans placed 500 mm away from the optic disc were averaged to perform the measurements. We measured the thickness of outer nuclear layer (ONL); outer plexiform layer (OPL); inner nuclear layer (INL); and a complex comprising the inner plexiform layer (IPL), ganglion cell layer (GCL), and nerve fiber layer (NFL) that we called IPL+GCL+NFL.

### 4.4. Full Field Electroretinogram

After overnight dark adaptation, mice were anesthetized with ketamine (80 mg/kg) and xylazine (8 mg/kg). Eye drops were used to dilate the pupils (0.5% mydriaticum, 5% neosynephrine). Body temperature was maintained at 37 °C through the use of a heating pad. Contact lens electrodes for mice (Mayo Corporation, Aichi, Japan) were placed on the corneal surface to record the full field electroretinogram (ERG). A needle electrode placed subcutaneously in the forehead served as reference, and a needle electrode placed in the back served as ground. Recordings were made from both eyes simultaneously. Stimulus presentation and data acquisition were provided by the Espion E2 system (Diagnosys LLC, Lowell, MA, USA). Eight levels of stimulus intensity ranging from 0.0003 cd.s/m^2^ to 30 cd.s/m^2^ were used for the dark-adapted ERG recording. Each scotopic ERG response represents the average of five responses from a set of five flashes of stimulation. To isolate cone responses, 20 min of light adaptation at 20 cd/m^2^ was performed to saturate rod photoreceptors. For the light-adapted ERG recording, a stimulus intensity of 3 cd.s/m^2^ was recorded on the same rod pathway-suppressive white background as for the light adaptation. Each cone photopic ERG response represents the average of twenty responses to a set of twenty consecutive flashes.

### 4.5. Optomotor Response

Since the structure and function of the *Gpr179*^+/+^ and *Gpr179*^+/−^ mice retinas were not distinguishable, we continued the experimentations only on *Gpr179*^+/+^ and *Gpr179*^−/−^ mice. The optomotor test was performed as previously described [43]. After overnight dark adaptation, mice were placed on a grid platform (11.5 cm diameter, 19 cm above the bottom of the drum) surrounded by a motorized drum (29 cm diameter) that could be revolved clockwise or anticlockwise at two revolutions per minute. Vertical black and white stripes of defined spatial frequency were presented to the animal. Spatial frequencies tested were 0.063, 0.125, 0.25, 0.5, and 0.75 cycles per degree (cpd). The stripes were rotated for 1 min in each direction with an interval of 10 s between the two rotations. Animals were video-taped using a digital video camera for subsequent scoring of head movements. Tests were initially performed under scotopic conditions, using the night shot function of the camera. Mice were then subjected to two lamps of 60 Watts each for 5 min, and photopic measurements were performed. Head movements were scored only if the angular speed of the movement corresponded to that of the drum rotation. Head movements in both directions were averaged to obtain the number of head movements per minute.

### 4.6. Immunohistochemistry

#### 4.6.1. Preparation of Retinal Sections for Immunohistochemistry

Mice were killed by CO_2_ administration and cervical dislocation. Eyes were removed and prepared following three methods of fixation. For method 1, the anterior segment and lens were removed, and the eyecup was fixed in ice cold 4% (*w/v*) paraformaldehyde in 0.12 M phosphate buffer, pH 7.2 for 20 min. For method 2, we made a hole just behind the ora serrata and placed the eyeball in 4% (*w/v*) paraformaldehyde in 0.12 M phosphate buffer, pH 7.2 for 5 min at room temperature. We then removed the anterior segment, and the lens and the eyecup were again fixed for 20 min in paraformaldehyde at room temperature. For method 3, we used the same protocol than method 2, but 5 min and 20 min of fixation were performed in ice cold 4% (*w/v*) paraformaldehyde in 0.12 M phosphate buffer, pH 7.2. After fixation, the eyecups were washed three times in ice-cold PBS and cryoprotected with increasing concentrations of ice-cold sucrose in 0.12 M phosphate buffer, pH 7.2 (10% and 20% for 1 h each and 30% overnight). Finally, the eyecups were embedded in 7.5% gelatin–10% sucrose and frozen in a dry ice-cooled isopentane bath. Sections were cut at a thickness of 20 µm on a cryostat and mounted onto glass slides (Super-Frost, Thermo Fisher Scientific, Waltham, MA, USA). The slides were air dried and stored at −80 °C.

#### 4.6.2. Immunostaining of Retinal Cryosections

Primary antibodies used for immunostaining are listed in Table 1. TRPM1 antibody was a generous gift from Dr Kirill Martemyanov (The Scripps Institute, Jupiter, FL, USA). Immunohistochemistry on retinal sections was performed following two protocols depending on the primary antibody we used (Table 1). For method 1, sections were blocked by incubation at room temperature for 60 min in 10% (*v/v*) donkey serum and 0.3% (*v/v*) Triton X-100 in PBS. Subsequently, the sections were incubated with primary antibodies in blocking solution overnight at room temperature. After washing in PBS, the sections (Table 1) were incubated with secondary antibodies coupled to Alexa Fluor 488 or Cy3 (Jackson ImmunoResearch, West Grove, PA, USA) at a dilution of 1:1000 in PBS for 1.5 h at room temperature. The slides were stained with DAPI and subsequently cover-slipped with mounting medium (Mowiol, Merck Millipore, Billerica, MA, USA). For method 2, sections were blocked by incubation at room temperature for 60 min in 0.2% (*w/v*) gelatin and 0.25% (*v*/*v*) Triton X-100 in PBS. Subsequently, the sections were incubated with primary antibodies (Table 1) in blocking solution overnight at room temperature. After washing in 0.1% (*v/v*) Triton X-100 in PBS, the sections were incubated with secondary antibodies coupled to Alexa Fluor 488 or Cy3 (Jackson ImmunoResearch, West Grove, PA, USA) at a dilution of 1:1000 in the washing solution for 1.5 h at room temperature. The slides were stained with DAPI and subsequently cover-slipped with mounting medium (Mowiol, Merck Millipore). None of the secondary antibodies used gave significant staining when used without primary antibodies (data not shown).

#### 4.6.3. Image Acquisition

Fluorescent staining signals were captured with a confocal microscope (FV1000, Olympus, Hamburg, Germany) equipped with 405, 488, and 559 nm lasers. Confocal images were acquired with a 40x objective compatible with oil (lens NA: 1.3) imaging pixels of 310 nm and 77 nm in width and height for zoom 1 and 4, respectively, and using a 0.52 µm step size. Each image corresponds to the projection of three optical sections. For figures, brightness and contrast were optimized (ImageJ, version 1.49; National Institutes of Health, Bethesda, MD, USA).

### 4.7. Statistical Analyses

Statistical analyses were performed using SPSS Statistics (version 19.0, IBM, Armonk, NY, USA). Post hoc comparisons were used to compare the genotypes two by two when the Kruskal–Wallis test permitted rejecting the hypothesis H0. The number of animals used for the different phenotyping experiments and groups is described above (Animal Care). Results were considered statistically significantly different if *p* < 0.05.

## Figures and Tables

**Table 1:** Primary antibodies used in immunohistochemistry in this study.

Antibody	Species	Dilution	Reference	Retinal Sections Preparation	Immunohistochemistry
GNB5	rabbit	1:300	ABIN1451282 (Antibodies Online, Aachen, Germany)	Method 2	Method 2
GPR179	mouse	1:200	Ab-887-YOM (Primm, Milan, Italy)	Either Method 1 or Method 3	Method 1
GRM6	guinea pig	1:200	AP20134SU-N (Acris, Herford, Germany)	Method 1	Method 1
Lectin PNA 488 conjugate	*Arachis hypogaea*	1:1000	L21409 (Life Technologies, ThermoFisher Scientific, Waltham, MA, USA)	Method 1	Method 1
LRIT3	rabbit	1:500	Neuillé et al., 2015 [8]	Method 1	Method 2
PKCα	mouse	1:200	P5704 (Sigma-Aldrich, Saint Louis, MI, USA)	Method 3	Method 1
RGS7	rabbit	1:250	sc-28836 (Santa-Cruz)	Method 2	Method 2
RGS11	goat	1:200	sc-9725 (Santa-Cruz, Dallas, TX, USA)	Method 2	Method 2
TRPM1	sheep	1:500	Cao et al., 2011 [66]	Method 1	Method 2

## Data Availability

Not applicable.
